# Supplementary material for: Forecasting extremely high ischemic stroke incidence using meteorological time serie
Source: PLoS One. 2024 Sep 11;19(9):e0310018. doi: 10.1371/journal.pone.0310018 (PMC11389912; doi:10.1371/journal.pone.0310018)
Supplement: S1 File — The zip file contains RNotebook source code, data, and html report with detailed results of data analyses, for each district in a separate subdirectory. READ .ME file contains instructions for reproducing the results. (ZIP) [file pone.0310018.s001.zip › _R_share/_MT/report_MT.html]

report\_MT.R


# report\_MT.R

#### mg

#### 2023-09-13

# Martin

# EDA of stroke counts ts

## Descriptive statistics

| rok | n | mean | sd | min | Q1 | median | Q3 | max | percZero | total\_number\_of\_strokes |
| --- | --- | --- | --- | --- | --- | --- | --- | --- | --- | --- |
| 2015 | 358 | 0.49 | 0.73 | 0 | 0 | 0 | 1 | 4 | 63.13 | 174 |
| 2016 | 366 | 0.56 | 0.80 | 0 | 0 | 0 | 1 | 5 | 59.56 | 205 |
| 2017 | 365 | 0.55 | 0.77 | 0 | 0 | 0 | 1 | 5 | 57.81 | 200 |
| 2018 | 365 | 0.53 | 0.70 | 0 | 0 | 0 | 1 | 3 | 57.53 | 194 |
| 2019 | 364 | 0.51 | 0.73 | 0 | 0 | 0 | 1 | 4 | 60.44 | 184 |

## Time series plot

## Barplot of stroke counts frequency

### 90th percentile of stroke counts; year by year

```
  year percentile_90
1 2015             1
2 2016             2
3 2017             1
4 2018             1
5 2019             1
```

## Lag plot of stroke counts ts

## Lag crossplot of stroke counts ts and t\_max ts

# EDA of roll-sum smoothed and detrended stroke counts ts

## Time series plot of roll-sum smoothed and detrended stroke counts

## Lag plot of smoothed & detrended stroke counts ts

## Lag crossplot of smoothed detrended stroke counts ts and smoothed t\_max ts

## Density plot of smoothed detrended stroke counts

# EDA of roll-mean smoothed weather ts

## Time series plot of roll-mean smoothed weather ts

# CCF for the smoothed, detrended stroke counts vs mean-smoothed weather

# EDA of extreme (Ext) ts

| extreme | Freq |
| --- | --- |
| 0 | 1636 |
| 1 | 182 |

# CCF for smoothed, detrended, binarized stroke counts (Ext) vs mean-smoothed weather

# Multivariate logistic regression model of Ext (full time span)

## AIC model selection

|  |  |
| --- | --- |
| Observations | 1815 |
| Dependent variable | Ext |
| Type | Generalized linear model |
| Family | binomial |
| Link | logit |

|  |  |
| --- | --- |
| 𝛘²(21) | 473.58 |
| Pseudo-R² (Cragg-Uhler) | 0.48 |
| Pseudo-R² (McFadden) | 0.40 |
| AIC | 752.67 |
| BIC | 873.75 |

|  | exp(Est.) | 2.5% | 97.5% | z val. | p |
| --- | --- | --- | --- | --- | --- |
| (Intercept) | 0.03 | 0.02 | 0.04 | -23.13 | 0.00 |
| tmin1 | 2.52 | 1.18 | 5.39 | 2.39 | 0.02 |
| tmin2 | 0.52 | 0.24 | 1.16 | -1.59 | 0.11 |
| tampl1 | 1.75 | 1.08 | 2.84 | 2.28 | 0.02 |
| tampl2 | 0.64 | 0.38 | 1.05 | -1.76 | 0.08 |
| press1 | 0.12 | 0.04 | 0.43 | -3.31 | 0.00 |
| press2 | 33.58 | 4.33 | 260.33 | 3.36 | 0.00 |
| press3 | 0.21 | 0.07 | 0.66 | -2.68 | 0.01 |
| hum1 | 1.43 | 1.17 | 1.74 | 3.47 | 0.00 |
| hum2 | 0.60 | 0.43 | 0.84 | -2.96 | 0.00 |
| hum3 | 1.21 | 0.99 | 1.47 | 1.90 | 0.06 |
| wind2 | 0.05 | 0.00 | 0.90 | -2.03 | 0.04 |
| wind3 | 8.27 | 0.53 | 129.93 | 1.50 | 0.13 |
| rain2 | 0.67 | 0.50 | 0.90 | -2.70 | 0.01 |
| rain3 | 1.24 | 0.94 | 1.64 | 1.56 | 0.12 |
| wci2 | 2.55 | 1.19 | 5.47 | 2.40 | 0.02 |
| wci3 | 0.49 | 0.24 | 1.03 | -1.88 | 0.06 |
| etv2 | 0.20 | 0.06 | 0.73 | -2.45 | 0.01 |
| etv3 | 3.32 | 0.98 | 11.26 | 1.92 | 0.05 |
| Ext1 | 29.80 | 17.82 | 49.84 | 12.94 | 0.00 |
| Ext2 | 2.75 | 1.58 | 4.79 | 3.56 | 0.00 |
| year | 0.86 | 0.72 | 1.03 | -1.66 | 0.10 |
|  |
| --- |
| Standard errors: MLE; Continuous predictors are mean-centered. |

## Forestplot

# Random Forest for Time Series of Ext (full time span)

## Top10 predictors, by importance

# Sequential forecasting study

## Multivariate logistic regression (GLM)

### Forecast time series

### True values

### In one plot

### Youden index

```
Method: maximize_metric 
Predictor: x 
Outcome: class 
Direction: >= 

    AUC    n n_pos n_neg
 0.6039 1318   138  1180

 optimal_cutpoint youden    acc sensitivity specificity tp fn  fp  tn
           0.0446 0.1846 0.6252      0.5507      0.6339 76 62 432 748

Predictor summary: 
    Data         Min.          5%    1st Qu.     Median      Mean    3rd Qu.
 Overall 0.0004171256 0.005332907 0.01655497 0.03353263 0.1105988 0.07028943
       0 0.0004171256 0.005215833 0.01604562 0.03218173 0.1078817 0.06792142
       1 0.0012792641 0.006988003 0.02465673 0.05077434 0.1338324 0.11648075
       95%      Max.        SD NAs
 0.7532306 0.9777909 0.2131382   0
 0.7523643 0.9777909 0.2119993   0
 0.8031017 0.9210117 0.2220785   0
```

### ROC

## Random Forest for Time Series

### Forecast time series

### In one plot

### Youden index

```
Method: maximize_metric 
Predictor: x 
Outcome: class 
Direction: <= 

   AUC    n n_pos n_neg
 0.568 1317   148  1169

 optimal_cutpoint youden    acc sensitivity specificity tp fn  fp  tn
           0.9385 0.1315 0.5748      0.5541      0.5774 82 66 494 675

Predictor summary: 
    Data      Min.        5%   1st Qu.    Median      Mean   3rd Qu.       95%
 Overall 0.2446786 0.4497822 0.8779548 0.9479429 0.8834278 0.9732548 0.9921040
       0 0.2446786 0.4342176 0.8833675 0.9501421 0.8858955 0.9737698 0.9920438
       1 0.3417873 0.5355758 0.7953312 0.9326063 0.8639367 0.9662431 0.9914390
   Max.        SD NAs
 1.0000 0.1589789   0
 1.0000 0.1599914   0
 0.9975 0.1498303   0
```

### ROC

## Croston’s forecasting method

## Sequential 1-step-ahead forecasting

## w = 0.5

### Forecast time series

### True values

### In one plot

### Youden index

```
Method: maximize_metric 
Predictor: x 
Outcome: class 
Direction: <= 

    AUC    n n_pos n_neg
 0.6441 1317   148  1169

 optimal_cutpoint youden    acc sensitivity specificity  tp fn  fp  tn
           0.4134 0.3097 0.5444      0.7973      0.5124 118 30 570 599

Predictor summary: 
    Data       Min.         5%    1st Qu.    Median      Mean   3rd Qu.
 Overall 0.01758862 0.03520963 0.12790659 0.3634399 0.3973966 0.5848391
       0 0.02039242 0.03520963 0.13552633 0.4150741 0.4143302 0.5866465
       1 0.01758862 0.03599378 0.08042094 0.1862585 0.2636448 0.3700598
       95%      Max.        SD NAs
 0.8599712 0.9788845 0.2847010   0
 0.8599712 0.9788845 0.2859864   0
 0.7669430 0.9788845 0.2355547   0
```

### ROC

## Comparing ROCs of GLM, RFTS and Croston’s method

## Bootstrap pairwise comparison of AUCs (for paired ROCs)

| comparison | pval | padj |
| --- | --- | --- |
| rf vs glm | 0.146 | 0.219 |
| rf vs cr | 0.041 | 0.124 |
| glm vs cr | 0.248 | 0.248 |

# Summary

**Findings**

The logistic regression (GLM) model in the sequential one-step-ahead
forecasting achieved sensitivity 0.551 and specificity 0.634 The values
were obtained for cutoff set to the Youden index cutoff.

RFTS attained sensitivity 0.554 and specificity 0.577, at the Youden
index cutoff.

Croston with w = 0.5: sensitivity 0.797, specificity 0.512 at the
Youden index cutoff.

# Session info

```
─ Session info ───────────────────────────────────────────────────────────────
 setting  value
 version  R version 4.0.5 (2021-03-31)
 os       CentOS Stream 8
 system   x86_64, linux-gnu
 ui       X11
 language (EN)
 collate  en_US.UTF-8
 ctype    en_US.UTF-8
 tz       Europe/Bratislava
 date     2023-09-13
 pandoc   2.19.2 @ /usr/lib/rstudio/resources/app/bin/quarto/bin/tools/ (via rmarkdown)

─ Packages ───────────────────────────────────────────────────────────────────
 package          * version   date (UTC) lib source
 cutpointr        * 1.1.1     2021-06-29 [1] CRAN (R 4.0.5)
 dplyr            * 1.1.2     2023-04-20 [1] CRAN (R 4.0.5)
 dygraphs         * 1.1.1.6   2018-07-11 [1] CRAN (R 4.0.5)
 flextable        * 0.7.1.010 2022-05-06 [1] Github (davidgohel/flextable@42fabfd)
 forecast         * 8.14      2021-03-11 [1] CRAN (R 4.0.5)
 FSA              * 0.9.4     2023-02-01 [1] CRAN (R 4.0.5)
 ggplot2          * 3.4.2     2023-04-03 [1] CRAN (R 4.0.5)
 ggpubr           * 0.4.0.999 2021-07-17 [1] Github (kassambara/ggpubr@ac5a01f)
 ggtext           * 0.1.1     2020-12-17 [1] CRAN (R 4.0.5)
 greybox          * 1.0.1     2021-09-22 [1] CRAN (R 4.0.5)
 InformationValue * 1.2.3     2016-10-30 [1] CRAN (R 4.0.5)
 jtools           * 2.1.3     2021-03-12 [1] CRAN (R 4.0.5)
 kableExtra       * 1.3.4     2021-02-20 [1] CRAN (R 4.0.5)
 knitr            * 1.42      2023-01-25 [1] CRAN (R 4.0.5)
 lmtest           * 0.9-38    2020-09-09 [1] CRAN (R 4.0.5)
 lubridate        * 1.9.0     2022-11-06 [1] CRAN (R 4.0.5)
 MAPA             * 2.0.4     2018-01-05 [1] CRAN (R 4.0.5)
 patchwork        * 1.1.1     2020-12-17 [1] CRAN (R 4.0.5)
 pROC             * 1.17.0.1  2021-01-13 [1] CRAN (R 4.0.5)
 rangerts         * 0.0.3     2022-02-19 [1] Github (hyanworkspace/rangerts@d0dd280)
 RColorBrewer     * 1.1-3     2022-04-03 [1] CRAN (R 4.0.5)
 roll             * 1.1.6     2020-07-13 [1] CRAN (R 4.0.5)
 scales           * 1.2.1     2022-08-20 [1] CRAN (R 4.0.5)
 sjPlot           * 2.8.12    2022-12-13 [1] Github (strengejacke/sjPlot@4058580)
 smooth           * 3.1.3     2021-09-22 [1] CRAN (R 4.0.5)
 timechange       * 0.1.1     2022-11-04 [1] CRAN (R 4.0.5)
 tsbox            * 0.3.1     2021-09-16 [1] CRAN (R 4.0.5)
 tsibble          * 1.1.1     2021-12-03 [1] CRAN (R 4.0.5)
 tsintermittent   * 1.9       2016-03-10 [1] CRAN (R 4.0.5)
 xts              * 0.12.1    2020-09-09 [1] CRAN (R 4.0.5)
 zoo              * 1.8-9     2021-03-09 [1] CRAN (R 4.0.5)

 [1] /home/mg/R/x86_64-pc-linux-gnu-library/4.0
 [2] /opt/R/4.0.5/lib/R/library

──────────────────────────────────────────────────────────────────────────────
```
