# Supplementary material for: Forecasting extremely high ischemic stroke incidence using meteorological time serie
Source: PLoS One. 2024 Sep 11;19(9):e0310018. doi: 10.1371/journal.pone.0310018 (PMC11389912; doi:10.1371/journal.pone.0310018)
Supplement: Attachment — Submitted filename: Response to Reviewers.docx [file pone.0310018.s002.docx]

Dear *Jyotir Moy Chatterjee*, M. Tech (CSE),

thank you very much for the care you gave to considering our manuscript *Forecasting extremely high ischemic stroke incidence using meteorological time series* and for selecting knowledgeable reviewers!

We have addressed the comments and suggestions in the revised version of the manuscript. Detailed response to reviewers can be found at the bottom of this document.

Concerning the Journal requirements:

1) In the revised version, we have reformatted Abstract so that it conforms with the PLOS ONE requirements. Supporting Information file, containing both the data and R code necessary for reproducing our analyses, was added as the Supporting Information. Also, we have used Pilcrow to indicate the authors who contributed equally.

2) Code sharing

We have included the R source code, data, and the resulting html reports as the Supporting Information S1_file.

3) Data Availability statement

We have included the data in the Supporting Information S1_file.

4) Please amend either the title on the online submission form (via Edit Submission) or the title in the manuscript so that they are identical.

5) We require you to either (1) present written permission from the copyright holder to publish these figures specifically under the CC BY 4.0 license, or (2) remove the figures from your submission.

In the revised version we have replaced Fig. 1 and Fig. 2. by equivalent ones, which are publicly available.

Thank you, again, for yours editorial work!

Best regards,

Vladimir Nosal, PhD, MD.

Martin, Dec 27, 2023

**Response to Reviewers**

**Reviewer #1**

Thank you for noting that figure numbering was missing in the manuscript. This was, in part, due to an error in Overleaf, which we have used for preparing the manuscript. It is corrected in the revised version.

**Reviewer #2**

The authors have used a number of ML methods to Forecast Extremely High Ischemic Stroke Incidence Using Meteorological Time Series

However there are some issues related to the paper.

There is no information about tuning the models, training and testing sets, uncertainties.

Also few meteorological data were used related to the Extremely High Ischemic Stroke Incidence. Extremely High Ischemic Stroke Incidence rate is also associated with air polution parameters which were ignored in this study.

Also it is not known why did the authors considered a binary response and have used logistic regression. They can use count response which is more informative. Age and sex were not considered. One thisg is that the used methods do not consider the spatial variations. According to the manuscript the authors have gathered the data from several regions therefore using spatio temporal would be more sound.

Thank you very much for the constructive criticism of our study! Below, we address yours question, and comments point by point:

- There is no information about tuning the models, training and testing sets, uncertainties.

Thank you for making this point!

RandomForest for Time Series was used with the default settings. There is no parameter to be tuned in Multivariate Logistic Regression. Croston’s forecasting method was used with the smoothing parameter *w* set to 0.5, which corresponds to the balance of the recent and past values.

We have added the following sentences to the text: 1) "*RFTS was used with the default settings.*", placed at the end of the first paragraph of Section 'Modelling study: Multivariate logistic regression and Random Forest for Time Series. 2) "*Croston's method was used with the value of the smoothing parameter set to 0.5, which corresponds to the balance of the recent and past values.*", placed at the end of the first paragraph of Section 'Forecasting study: GLM, RFTS and Croston’s method'.

As far as the training and testing is concerned, as we state in the paper (see Forecasting study: GLM, RFTS and Croston’s method), the time series of first 500 observations was used for the initial training. The forecasts were sequentially updated. We were interested in point forecasts and have quantified the quality of forecasts by the ROC curve.

- Also few meteorological data were used related to the Extremely High Ischemic Stroke Incidence. Extremely High Ischemic Stroke Incidence rate is also associated with air polution parameters which were ignored in this study.

Thank you for pointing out that other meteorological variables should also be considered. Unfortunately, we did not have access to the air pollution data.

- Also it is not known why did the authors considered a binary response and have used logistic regression. They can use count response which is more informative.

Thank you for the comments! Our motivation for turning the counts time series into binary time series is explained in the Introduction of the original submission, where we state:

"In the pursuit of making our approach clinically relevant, we chose to forecast extremely high stroke counts. This facet holds practical significance, enabling the adjustment of resources to accommodate the predicted surge in stroke cases. Notably, forecasting high stroke counts sidesteps the challenge of selecting a quality measure for predictions. By transforming raw stroke counts into binary time series (ordinary stroke count/high stroke count), we can employ sensitivity and specificity metrics to quantify forecast quality."

- Age and sex were not considered.

We are not aware of any method for relating together the scalar number of strokes and a vector of gender (or age) of the patients who had the stroke. To fix the ideas, let at day *d* and district *D* there be *k* patients with stroke. Sex of the patients is a vector of length *k;* say, (Female, Female, Male, Male, Male) assuming that *k* = 5. How could be the vector of gender used to predict the number of strokes, which is a scalar? The vector should first be turned into a scalar. But how? By averaging it? The other option would be to add gender vector as the *k* predictors. But, at each day there would be different number of columns in the design matrix. This is not viable, either.

- One thisg is that the used methods do not consider the spatial variations. According to the manuscript the authors have gathered the data from several regions therefore using spatio temporal would be more sound.

Again, many thanks for the suggestion! Spatiotemporal modeling would definitely be more encompassing, as the forecasts in one place could borrow strength from data in the other districts. This could be an interesting venue to follow in the future research. We have added it to the limitations of the study discussion.

The following paragraph was added to the Discussion:

In addressing the limitations of our study, the insightful feedback from an anonymous reviewer highlights the importance of considering air pollution alongside the meteorological variables we investigated, given its known association with stroke. Furthermore, our approach to forecasting time series could be enhanced by incorporating spatiotemporal methods. These valuable suggestions provide avenues for future research investigations.
