# Supplementary material for: Forecasting extremely high ischemic stroke incidence using meteorological time serie
Source: PLoS One. 2024 Sep 11;19(9):e0310018. doi: 10.1371/journal.pone.0310018 (PMC11389912; doi:10.1371/journal.pone.0310018)
Supplement: Attachment — Submitted filename: Response to Reviewers.docx [file pone.0310018.s003.docx]

Dear *Jyotir Moy Chatterjee*, M. Tech (CSE),

thank you, again, for the care you gave to considering our manuscript *Forecasting extremely high ischemic stroke incidence using meteorological time series* and for selecting knowledgeable reviewers!

We have addressed the comments and suggestions in the revised version of the manuscript. Detailed response to reviewers can be found at the bottom of this document.

Concerning the Journal requirements:

1) We have reviewed the reference list for its completeness and correctness. Upon a suggestion from Reviewer #4 we have added another reference (#44) to the References. The change is marked also in the *Revised Manuscript with Track Changes* pdf file.

Thank you, again, for yours editorial work!

Best regards,

Vladimir Nosal, PhD, MD.

Martin, Apr 9, 2024

**Response to Reviewers**

**Reviewer #4**

Prompted by your suggestion to add references that show the limited capability of weather factors to forecast incidence of hospital admissions for other diseases, we searched for such a literature. We did it for cardiovascular disease. The state of art is very similar to that of ischaemic stroke - all the papers that we have scrutinized, model association between weather factors and the response (event, or number of events) and report which of the weather factors are significant, together with quantification of risk of the event. We were not able to find a research reporting a forecasting performance of the models. This reinforces the main points that we make by this research:

1) many weather factors demonstrate statistically significant association with incidence of hospital admissions for a disease,

2) however, statistical significance not necessarily imply predictive/forecasting accuracy;

3) hence, it is of interest to assess the predictive power of the weather factors;

4) and to make it of practical interest, we decided to predict extremely high stroke counts so that adjustment of resources can be made to accommodate the predicted surge in stroke cases.

We add the above text to the Discussion section of the revised manuscript.

We agree with your point that there are numerous features relevant to acute stroke and we add the reference you have suggested to the manuscript. On technical level, however, it should be noted that the patient level data (such as gene expression, age, gender, etc.) cannot be reconciled with the counts data.

Thank you for your comments and suggestions!
