# Supplementary material for: Forecasting extremely high ischemic stroke incidence using meteorological time serie
Source: PLoS One. 2024 Sep 11;19(9):e0310018. doi: 10.1371/journal.pone.0310018 (PMC11389912; doi:10.1371/journal.pone.0310018)
Supplement: Attachment — Submitted filename: Letter_to_Editor__Aug10_2024.docx [file pone.0310018.s004.docx]

Dear *Jyotir Moy Chatterjee*, M. Tech (CSE),

thank you, again, for the care you gave to considering our manuscript *Forecasting extremely high ischemic stroke incidence using meteorological time series* and for yours comments and suggestions!

We have addressed the comments and suggestions in the revised version of the manuscript. Detailed response to the Additional Editor Comments can be found at the bottom of this document.

Concerning the Journal requirements:

1) We have reviewed the reference list for its completeness and correctness. Upon a suggestion from Reviewer #4 we have replaced reference (#44) by the one suggested by the Reviewer. The change is marked also in the *Revised Manuscript with Track Changes* pdf file.

Thank you, again, for yours editorial work!

Best regards,

Vladimir Nosal, PhD, MD.

Martin, Aug 10, 2024

**Response to Additional Editor Comments**

Thank you for your insightful comments and suggestions regarding the limitations of our study. We greatly appreciate the time and effort you put into reviewing our manuscript.

**1.** **Keywords are missing.**

Thank you for pointing this out. We checked the official PLoS ONE LaTeX template (https://journals.plos.org/plosone/s/latex) and noted that there is no option available for providing keywords. We also checked a couple recently published papers from PLoS ONE and no keywords are presented in the papers.

(https://journals.plos.org/plosone/article?id=10.1371/journal.pone.0307842

https://journals.plos.org/plosone/article?id=10.1371/journal.pone.0305825)

However, if keywords are needed for other purposes, we suggest the following: ischemic stroke; weather; time series forecasting;

**2. The main contribution of the work is not clear from the introduction.**

Thank you for pointing out that the main contribution of the work is not stated clearly.

Original formulation of the main contribution:

"We propose an augmentation of traditional association modeling through stroke count forecasting,

intending to achieve the following objectives:

i) Evaluate the predictive efficacy of different forecasting methods for stroke counts;

ii) Compare forecasts leveraging meteorological time series against those grounded solely in historical stroke count data."

was in the revised version reformulated into a more impactful form:

"With this perspective, in our study, we augment traditional association modeling by incorporating stroke count forecasting. Our primary contribution is twofold:

Evaluation of Predictive Methods: We systematically evaluate the predictive efficacy of different forecasting methods, including Generalized Linear Regression (GLM), RandomForest for Time Series, and Croston's method, in predicting stroke counts.

Comparison of Forecasting Approaches: We compare forecasts that leverage meteorological time series data against those based solely on historical stroke count data. This comparison aims to determine whether incorporating meteorological data improves the accuracy and utility of stroke count predictions."

**3. The overall organization of the paper is not presented in the introduction section.**

Thank you for making the point! We checked the above-mentioned two papers recently published by PLoS ONE, and they do not contain paper organization in the introduction section.

**4. The study is based on data from stroke centers in the Slovak Republic, which may not be representative of other regions with different climates or healthcare systems.**

Thank you for this important comment. We acknowledge the regional specificity of our data, but we would like to emphasize that the stroke centers in our study cover all four climate types present in Slovakia, as classified by the Köppen-Geiger system. This diversity within a single country provides a broad range of meteorological conditions, which could offer insights applicable to other regions with similar climates.

**5. The five-year period of data collection might not capture long-term trends or rare events that could affect stroke incidence.**

Thank you for your comment. We recognize that a longer dataset could potentially capture more rare events or long-term trends. However, given the constraints of available data, our study focuses on exploring patterns and associations within the five-year timeframe.

**6. Findings may not be applicable to populations outside the Slovak Republic, particularly those in regions with significantly different meteorological conditions.**

Thank you for highlighting this limitation. We have added a statement in the revised manuscript to acknowledge that our findings may be most relevant to regions with similar climatic conditions to Slovakia and may not be directly applicable to regions with significantly different meteorological conditions.

**7. The quality and consistency of the meteorological and stroke data across different centers might vary, potentially introducing bias.**

Regarding the meteorological data, we utilized official datasets from national meteorological services, which are consistent and standardized across the country. However, we acknowledge that there could be variations in stroke data collection practices across different centers. However, given that the selected stroke centers are the major one, we are rather confident that the data collection practices are homogeneous across the centers.

**8. The study may not adequately account for other factors influencing stroke incidence, such as air pollution, socioeconomic status, or healthcare access.**

Thank you for this insight. We have addressed the issue of air pollution in a previous revision. As for healthcare access, the selected stroke centers are major hospitals located in urban areas, minimizing the potential bias related to healthcare accessibility. However, we acknowledge that other factors such as socioeconomic status were not included in our study, which we now explicitly state as a limitation.

**9. By concentrating on extreme stroke events (90th percentile), the study may overlook insights into more common stroke occurrences, which could provide a broader understanding.**

Thank you for this observation. Initially, our study focused on forecasting raw stroke counts, but the results indicated that meteorological data did not significantly improve forecasting accuracy. To enhance the clinical relevance of our predictions, we shifted our focus to extreme stroke events, which are critical for resource allocation and management. While this approach may overlook more common occurrences, it allows us to address specific, high-impact scenarios in healthcare.

**10. The transformation of stroke counts into a binary outcome (high vs. ordinary) might oversimplify the complex nature of stroke incidence, potentially missing subtler trends.**

We understand the concern about potential oversimplification. However, as explained in response to point 9, our binary approach was motivated by the need for practical and interpretable predictions in a clinical setting. This approach facilitates the use of sensitivity and specificity metrics, which are more intuitive for clinicians compared to other forecasting metrics such as the Symmetric Mean Squared Error that are used to quantify forecasting accurracy in count time series.

**11. The limited predictive accuracy of all three methods suggests that the models may not be well-suited for forecasting extreme stroke events, indicating a need for alternative approaches.**

We agree that exploring alternative forecasting methods could be valuable. Our study used Generalized Linear Regression, Random Forest for Time Series, and Croston's method as a starting point. We have made our data publicly available, inviting other researchers to test different approaches that may improve predictive accuracy.

**12. Croston’s method, which relies solely on historical stroke data, may not capture the influence of changing meteorological conditions over time.**

Indeed, Croston's method was used as a baseline reference due to its univariate nature. While it does not incorporate external factors like meteorological data, it provides a useful benchmark against which to measure the performance of more complex models that do incorporate such data.

**13. The use of multivariate logistic regression and Random Forest models could lead to overfitting, particularly if the models are overly complex relative to the size of the dataset.**

We acknowledge the risk of overfitting. To mitigate this, we used validation techniques to ensure that the model's performance was evaluated on unseen data.

**14. The study may not have identified the most relevant meteorological variables influencing stroke incidence, leading to weak correlations.**

In selecting relevant meteorological variables, we used the Akaike Information Criterion (AIC) for the GLM and the Variable Importance algorithm for Random Forest. These methods are well-established for feature selection, and we are confident that they have identified the most relevant variables influencing stroke incidence within the dataset available to us.

**15. The daily resolution of meteorological data might be insufficient to capture finer-scale weather patterns that could impact stroke risk.**

Thank you for raising this point. We acknowledge that finer temporal resolution could reveal additional insights. However, stroke data at finer resolutions (e.g., hourly) are not available in Slovakia. Our study, therefore, used the best available data to explore the relationship between daily meteorological conditions and stroke incidence.

**16. The study does not appear to consider potential temporal lags between changes in weather conditions and stroke incidence, which could affect the accuracy of the predictions.**

We ***have*** accounted for temporal lags in our analysis. Formula (1) in the manuscript outlines the inclusion of lagged weather time series in our models, both for the Generalized Linear Model and Random Forest for Time Series. This ensures that delayed effects of weather on stroke incidence are considered in our predictions.

**17. The complexity of Random Forest and logistic regression models may make it difficult to interpret the relationship between specific meteorological factors and stroke incidence.**

We agree that the interpretability of Random Forest models can be challenging. However, the logistic regression model offers interpretable odds ratios, which we have used to discuss the impact of specific meteorological factors on stroke incidence. We acknowledge the trade-off between model complexity and interpretability and have aimed to balance these considerations in our analysis.

**18. The models were developed and tested on the same dataset, which may limit the validity of the findings when applied to other datasets or populations.**

This is true for our modeling study. However, in the sequential forecasting study, we employed a walk-forward validation approach, where models were trained on data up to time

t and then used to forecast for time t+1. This methodology helps to mitigate the issue of overfitting to a specific dataset, providing a more robust evaluation of the model's predictive capabilities.

**19. While the study discusses seasonality, the forecasting models do not explicitly incorporate seasonal patterns, which could be a significant factor in stroke incidence.**

The absence of explicit seasonality incorporation in the models is due to the lack of clear seasonal patterns in the data. We conducted an exploratory analysis to detect seasonality, but it was not sufficiently pronounced to justify its inclusion in the models.

**20. The study focuses on meteorological factors and may overlook other critical factors, such as lifestyle changes, that could influence stroke incidence.**

We agree that factors such as lifestyle changes could influence stroke incidence. However, our study focuses on the relationship between weather conditions and stroke incidence within a specific five-year period. During this time, no significant lifestyle changes were reported in Slovakia, which is why they were not included in our models.

**21. The number of extreme stroke events (surpassing the 90th percentile) may be small, leading to challenges in building robust predictive models.**

We acknowledge that the small number of extreme events poses a challenge for model robustness. This limitation is inherent to studying rare events, but we believe that focusing on these extreme cases provides valuable insights, particularly for healthcare planning and resource allocation.

**22. The binary prediction approach may result in a high rate of false positives or false negatives, reducing the practical utility of the forecasts.**

Thank you for this observation. The binary classification approach was chosen to simplify the interpretation of results and make the forecasts more actionable in a clinical setting. We agree that a high rate of false positives or false negatives could reduce the utility of the forecasts. Our analysis shows that meteorological data alone do not provide strong predictive information for extreme stroke events, which contributes to the observed rates of misclassification. This highlights the need for further research to identify additional factors that may improve prediction accuracy.

**23. Using predictive models to forecast extreme medical events could raise ethical concerns, particularly if the forecasts are not accurate enough to inform effective interventions.**

Thank you for bringing up this concern. The primary objective of our study is to develop predictive models that can forecast extremely high stroke counts, thereby aiding clinic administrators in better allocating human resources, such as clinicians, to meet potential surges in demand. The use of these forecasts is intended for logistical and planning purposes, not for making direct clinical decisions about individual patient care. Therefore, we believe that the ethical risks are minimal in this context. Our forecasts are designed to inform staffing and resource management, ensuring that clinics are better prepared for potential increases in stroke cases, rather than guiding treatment decisions. We have clarified this objective and its implications in the revised manuscript.

---

***Thank you once again for your insightful comments and suggestions regarding the limitations of our study. We greatly appreciate the time and effort you put into reviewing our manuscript. In response to your feedback, we have carefully revised the Discussion section to incorporate most of the limitations you pointed out. These additions have helped us provide a more balanced and comprehensive evaluation of our findings, ensuring that the manuscript more accurately reflects the scope and implications of our study.***
